# Aging, Natural Bioactive Compounds and Non-Communicable Chronic Degenerative Diseases

**DOI:** 10.3390/nu17091442

**Published:** 2025-04-25

**Authors:** Annalisa Noce, Nicola Di Daniele

**Affiliations:** 1 Department of Systems Medicine, University of Rome Tor Vergata, 00133 Rome, Italy; didaniele@med.uniroma2.it; 2 UOSD Nephrology and Dialysis, Policlinico Tor Vergata, 00133 Rome, Italy; 3 Fondazione Leonardo per le Scienze Mediche Onlus, Policlinico Abano, 35031 Abano Terme, Italy

In recent decades, the world has witnessed a major demographic change characterized by an aging of the general population. Across the globe, the number of people aged 65 and over is increasing enormously; between 1990 and 2017, the percentage of elderly people increased globally from 6.1% to 8.8%, and it is estimated that their number will reach approximately 1.5 billion (i.e., 16% of the world population) by 2050 [1,2]. The aging of the world’s population could have enormous repercussions on Health Systems due to the numerous age-related chronic diseases [3,4,5,6]. Although aging is a physiological process, it is closely related to the increased risk of non-communicable chronic degenerative disease (NCCDD) onset, such as diabetes mellitus, arterial hypertension, chronic kidney disease (CKD), neurodegenerative diseases, cancers, chronic respiratory diseases, and cardiovascular diseases [7,8]. There are multiple biological mechanisms underlying the predisposition for these ailments, including increases in oxidative stress and the chronic inflammatory state [9,10].

Numerous recent studies have aimed at investigating new possible strategies to counteract the aging and the onset of NCCDDs. Many have focused on the use of adjuvant treatments, free of side effects, based on natural bioactive compound (NBC) consumption. NBCs are molecules of various nature and origin, mainly contained in plant-based foods, capable of exerting several beneficial effects on human health [11,12,13]. Specifically, they can perform important anti-aging actions; for example, they are able to delay the aging and reduce the risk of aging-related diseases, prolonging lifespan and promoting the well-being of the individual through the regulation of various physiological processes [14,15,16,17,18,19,20].

In our Special Issue, we explore, through reviews and original articles, this interesting field.

Di Lauro et al. (contribution 1) examined the close bidirectional correlation between the gut microbiota alterations and the migraine onset, involving the gut–brain axis, and how this axis can influence the NCCDDs risk. The Authors highlighted the importance of proper nutrition, the consumption of specific NBCs, and lifestyle in order to combat migraines in NCCDDs patients. In the same context, Derkaczew et al. (contribution 2) studied new possible adjuvant treatments for neurodegenerative diseases (such as Alzheimer’s disease, Parkinson’s disease, and Huntington’s disease) based on myo-inositol and its derivatives. The Authors concluded that dietary supplementation with myo-inositol exhibited a promising effect in the treatment of these neurodegenerative disorders and could be a valid support for alleviating the depressive symptoms associated with them. Furthermore, the neuroprotective and anti-neuroinflammatory effect of the metabolites of the plant *Allium hookeri*, a member of the genus Allium, was also demonstrated by the study conducted by Doan et al. (contribution 3). Specifically, the Authors used a non-targeted metabolomics approach to comprehensively profile the chemical composition of metabolites in *A. hookeri*. The Authors emphasized N-trans-feruloyltyramine as the most potent neuroprotective compound of the studied plant, able to mitigate senescence-associated neurological diseases. In addition, Yoon et al. (contribution 4) examined the potential therapeutic properties of fermented ginseng berry extract as adjuvant treatment for Alzheimer’s disease. The Authors highlighted how high levels of active ginsenosides in fermented ginseng exert neuroprotective effects and improve cognitive functions through antioxidant properties and acetylcholinesterase inhibitory activities. Moreover, Zhou et al. (contribution 5) evaluated the mechanism of the anti-hypoxic neuroprotective effect of chebulic acid, isolated from Terminalia chebula, both in vitro and in vivo. In vitro results showed that chebulic acid seems to protect against oxygen–glucose deprivation/reoxygenation-induced neurotoxicity in SH-SY5Y cells, through an important indirect antioxidant action; in vivo results showed, instead, that treatment with chebulic acid determines a significant decrease in ischemic infarct volume and an improvement in motor performance in mice at 24 h after stroke. The study, therefore, lays the foundation for a possible new chebulic acid-based drug for the control of ischemic stroke in humans.

With regard to cardiovascular risk, Marrone et al. (contribution 6) investigated the cardioprotective effects of minor polar compounds (such as oleocanthal, oleacin, tyrosol, and hydroxytyrosol) in extra virgin olive oil (EVOO) on CKD patients. Specifically, the Authors revealed, for the first time, important anti-inflammatory effects and significant improvements in lipid and purine metabolism, atherogenic indices, and body composition, exerted by the minor polar compounds of EVOO in nephropathic patients.

Ranogajec et al. (contribution 7) explored the effects of NBCs consumption, contained in plant-based foods, on the improvement of lung function in patients with chronic obstructive pulmonary disease (COPD). In particular, the Authors estimated the dietary phytochemical index (DPI) and revealed the DPI’s association with the improvement of lung function, strength, and function of inspiratory muscles and peripheral muscles in COPD patients. The Authors concluded that a higher intake of phytochemicals could be useful as dietary intervention in COPD treatment.

Finally, Wu et al. (contribution 8) focused their study on the role of the Mediterranean diet, which encourages a high consumption of plant-based foods, in promoting healthy aging. In particular, the Authors analyzed dietary data from over 7000 subjects, highlighting how a higher adherence to the Mediterranean diet showed a positive correlation with levels of soluble Klotho, a marker related to aging. A lower odds ratio for aging was, thus, observed in subjects who had a higher adherence to the Mediterranean diet.

In light of the conclusions of all the Authors who brilliantly contributed to our Special Issue, it is possible to affirm that the use of NBCs, mainly contained in plant-based foods, represents an important adjuvant strategy to counteract the onset of age-related NCCDDs and to slow down their progression.
